# Future care for long-term cancer survivors: towards a new model

**DOI:** 10.1007/s12094-021-02696-5

**Published:** 2021-10-29

**Authors:** M. Provencio, N. Romero, J. Tabernero, R. Vera, D. V. Baz, A. Arraiza, C. Camps, E. Felip, P. Garrido, B. Gaspar, M. Llombart, A. López, I. Magallón, V. M. Ibáñez, J. M. Olmos, C. Mur, A. Navarro-Ruiz, A. Pastor, M. Peiró, J. Polo, Á. Rodríguez-Lescure

**Affiliations:** 1 Medical Oncology Department, Spanish Lung Cancer Group (GECP), Hospital Universitario Puerta de Hierro Majadahonda, Calle Manuel de Falla 1Majadahonda, 28222 Madrid, Spain; 2 Cardiology and Cardiac Surgery Clinical Management Unit, Hospital Virgen del Rocio, Sevilla, Spain; 3 Medical Oncology Department, Hospital Universitario Vall d’HebronVHIOEuropean Society for Medical Oncology (ESMO), Barcelona, Spain; 4 Medical Oncology Department, Complejo Hospitalario de NavarraServicio Navarro de la SaludSpanish Society of Medical Oncology (SEOM), Navarra, Spain; 5 Medical Oncology Department, Hospital Universitario Virgen Macarena, Andalusian Comprehensive Oncology Plan, Sevilla, Spain; 6 Health Programmes, Health Care, Osakidetza, País Vasco, Spain; 7 Medical Oncology Department, Hospital General Universitario de ValenciaUniversidad de Valencia CIBERONCECO Foundation, Valencia, Spain; 8 Institut d’Oncologia, Vall d’Hebron University Hospital, Spanish Society of Medical Oncology (SEOM), Barcelona, Spain; 9 Department of Medical Oncology, IRYCIS, Hospital Universitario Ramón y Cajal, Spanish Society of Medical Oncology (SEOM), Madrid, Spain; 10 Spanish Association of Lung Cancer Patients (AEACaP), Valencia, Spain; 11 Valencia Oncology Institute Foundation, Valencia, Spain; 12 Pharmacy, Navarra Health Service—Osasunbidea, Navarra, Spain; 13 Medical Oncology Department, Hospital Universitario Vall d’Hebron, Spanish Oncology Nursing Society (SEEO), Barcelona, Spain; 14 Professional Organization of the Ministry of Health, Madrid, Spain; 15 Senate Health Committee, Andalusian School of Public Health, Granada, Spain; 16 Clínicas CAT Barcelona, Spanish Society of Health Directors (SEDISA), Barcelona, Spain; 17 Hospital Pharmacy ServiceHospital General Universitario de ElcheSpanish Society of Hospital Pharmacy, Alicante, Spain; 18 Spanish Society of Family and Community Medicine (SEMFyC), Madrid, Spain; 19 Universitat Ramon Llull, ESADE, Barcelona, Spain; 20 Centro de Salud Casar de Cáceres, Spanish Society of Primary Care Physicians (SEMERGEN), Cáceres, Spain; 21 Medical Oncology Department, Hospital General Universitario de Elche, Spanish Society of Medical Oncology (SEOM), Alicante, Spain

**Keywords:** Long cancer survivor, LCS patient care, Multidisciplinary cancer care

## Abstract

**Purpose:**

The increase in the prevalence "long-term cancer survivor” (LCS) patients is expected to increase the cost of LCS care. The aim of this study was to obtain information that would allow to optimise the current model of health management in Spain to adapt it to one of efficient LCS patient care.

**Methods:**

This qualitative study was carried out using Delphi methodology. An advisory committee defined the criteria for participation, select the panel of experts, prepare the questionnaire, interpret the results and draft the final report.

**Results:**

232 people took part in the study (48 oncologists). Absolute consensus was reached in three of the proposed sections: oncological epidemiology, training of health professionals and ICT functions.

**Conclusion:**

The role of primary care in the clinical management of LCS patients needs to be upgraded, coordination with the oncologist and hospital care is essential. The funding model needs to be adapted to determine the funding conditions for new drugs and technologies.

## Introduction

Improvements in cancer screening, diagnosis, and treatments [1] have resulted in an increase in the "long-term cancer survivor” (LCS) population [2, 3]. These patients have unique needs and an increased risk of experiencing certain medical and psychosocial problems in their lifetime [4], many of which may appear at a later stage [5]. This, together with the need for vigilance in the event of recurrences or new malignancies [5], requires multidisciplinary care [2].

For the period 2010–2014, the CONCORD-3 report described a clear increase in global survival for all cancers [6]. In the US, where half of patients diagnosed with cancer survive at least 10 years [2], databases are available to facilitate the study of LCS patients [7], as well as specific programmes for their care and follow-up [8]. The EUROCARE-5 study, which analysed data from 107 European registries with over 10 million cancer patients, demonstrated an increase in relative survival over 5 years in all countries analysed [9]. In Italy, a 3% increase in the annual cancer survival rate has recently been reported [10].

Data from the Spanish Network of Cancer Registries (REDECAN) revealed the survival rate for men and women diagnosed with cancer in the period 2008–2013 [11] to be 61.7 and 55.3%, respectively. Currently, assuming a broad definition of LCS, it is estimated that there are 1.5 million such patients in Spain [12, 13], with 100,000 new cases each year [14]. The increase in the prevalence of these patients is expected to increase the cost of LCS care, although there are no estimates for this.

Against this background, the aim of this study was to obtain information, through the Delphi methodology, that would allow us to optimise the current model of health management in Spain to adapt it to one of efficient LCS patient care.

## Materials and methods

### Objectives

The purpose of this study was to: (1) Identify the points of agreement/disagreement regarding LCS care expectations and forecasts in Spain among the agents involved; (2) Analyse the differences between the responses relating to the "desired” and the "forecast (actual)" outcomes, and (3) Provide healthcare professionals with information regarding the expectations, forecasts and care challenges of LCS patients in Spain.

### Study design

#### Scientific committee and panel of experts

This project was carried out between June 2018 and March 2020, using Delphi methodology. In the early stages, the advisory committee was set up to define the criteria for participation, select the panel of experts, prepare the questionnaire, interpret the results and draft the final report.

The panel of experts consisted of specialists and affected persons representing various groups involved in health care, patient care, pharmaceutical care, care management and political/administrative management.

#### Generation of statements

The advisory committee identified areas of uncertainty regarding LCS care that should determine the structure of the questionnaire. Twelve thematic sections were created: (1) Oncological epidemiology; (2) Cancer care planning; (3) Care and care coordination model; (4) Clinical and care services; (5) Funding model; (6) Evaluation measures; (7) Training of health professionals; (8) LCS patient involvement; (9) Information and communications technology (ICT) functions; (10) Clinical and translational research; (11) Access to new therapies; and (12) Barriers to the transformation process.

The timeframe for the consultation was 10 years. The questions making up the questionnaire were divided into closed multiple-choice options, with the possibility of adding optional comments on each question. Questions were designed to be answered as an either/or (yes/no) response or by means of a Likert scale (5 and 4 levels of agreement in the first and second Delphi rounds, respectively).

#### Definition of a long-term survivor

To standardise the individual evaluation criteria for the survey responses, the advisory committee considered it necessary to include a definition of LCS that took into account the characteristics of each type of cancer. Participants were therefore asked to respond taking the following definition into account:

"Beyond the specific characteristics that might be deemed necessary with regard to the original type of cancer, once classified as long-term survivors, all these patients share common care needs. Needs that today could be similar to those of chronic patients. Throughout this questionnaire, questions are asked that will help to formulate a common cancer care model, at the initial stage, and that will undoubtedly need to be adapted according to the characteristics of each type of cancer in later phases. For this reason, we ask you to think of a general cancer patient who has increased his or her expected survival either disease-free or progression-free."

#### Dynamics of the Delphi study

Panel members completed two Delphi rounds across a platform. The end-of-consultation criterion for each question was consensus, defined as a degree of convergence of at least 80% of the individual estimates. After the first round, those questions with differing criteria passed to a second round, and were rewritten where wording warranted improvement. For the second consultation round, the overall results and the individual response that each participant had given in the first round were incorporated into the platform. In this way, each had the opportunity to reconsider their responses in light of the overall position of the panel of experts.

Once analysis of the results was available, the advisory committee met to formulate the study conclusions. It also addressed prioritising and identifying the challenges with a view to shaping the strategy for transformation of the LCS care model.

The final report was reviewed by independent experts to increase the validity of the Delphi study.

#### Analysis and interpretation of results

At the end of each consultation round, the responses to the questionnaires were analysed overall and by subgroups, in accordance with the following statistical parameters: number of responses (N) and percentage of total (%). The percentages of agreement were rounded up or down to whole numbers.

For the purposes of interpretation and graphical representation of the results, the responses were grouped as follows: (1) Unanimity: 100% agreement of the panel of experts with the claim; (2) Consensus: 80–99% agreement of the panel of experts; (3) Majority: 66–79% agreement; (4) Difference of opinion: agreement of fewer than 66% of the panel members.

To reduce possible biases due to the over-representation of some groups, differences of opinion of more than 20% between specialties were analysed.

The results of this study do not reflect the impact of the SARS CoV-2 (COVID 19) pandemic on the Spanish health system, something that must be taken into account for reading the results in their true context.

## Results

### Profile of the panel of experts

A total of 232 people took part in the first round of the study, of whom 79.7% (*n* = 185) also responded to the second round. Some 60% of those taking part were from the healthcare sector (health personnel and patients); the remainder were involved in health policy and management (28 and 8%, respectively). Within the healthcare field, the most represented groups were medical oncologists (21%) and primary care doctors who had experience with chronic patients (17%).

Of the total number of oncologists (*n* = 48), 54.16% were lung cancer specialists. A subanalysis of the responses obtained from this group with respect to the overall group of oncologists was carried out to mitigate a possible bias due to the overrepresentation of this group.

### Extent of overall agreement

**Fig. 1:**
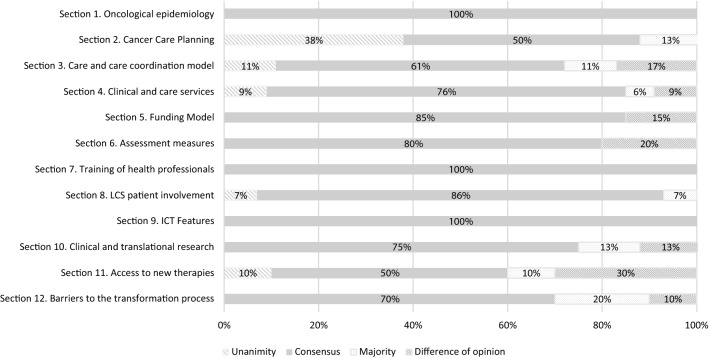
Level of agreement reached for each section in the Delphi study

The degree of agreement obtained was analysed based on thematic sections (Fig. 1). Absolute consensus was reached in three of the proposed sections: oncological epidemiology, training of health professionals and ICT functions. Conversely, the greatest differences of opinion were observed in the sections that addressed access to new therapies, evaluation measures and the care and coordination model.

In the first Delphi round, 51% consensus was achieved. The second round assessed the remaining statements, including those that were reworded, and there was then 64% consensus. Altogether 82% of the items were agreed on a consensus basis, 6% of them unanimously.

Table 1 shows the response rates to the statements and the degree of consensus reached. The most relevant results are summarised below.

**Table 1:** Percentage of responses to Delphi survey items, together with the level of agreement reached

Item	Result		Level agreement
**Section 1: oncological epidemiology**			
Factors that will have an impact on the increased prevalence of LCSs and on the Spanish National Health Service in the next 10 years	Yes, it will have an impact on		
1. Medical-therapeutic and technological improvements	**95%**		C
2. Changes in population lifestyles	**96%**		C
3. Public access to health services	**98%**		C
4. The coexistence of comorbidities will have an impact on the emergence of new cancer subpopulations that will increase the complexity of care	**92%**		C
**Section 2: cancer care planning**			
Cancer care planning forecasts for the next 10 years	Desired	Actual	
5. Oncology planning will address the need to correct territorial inequalities between communities through the appropriate allocation of the necessary resources	**Yes (98%)**	No (71%)	C/M
6. Oncology planning will address access to more personalised therapies	**Yes (100%)**	**Yes (91%)**	U/C
7. Oncology planning will be included in the care plans for LCSs and will take the increase in their prevalence into account	**Yes (100%)**	**Yes (90%)**	U/C
8. Oncology planning for LCS patient care will improve interaction between primary care and hospital care	**Yes (100%)**	**Yes (87%)**	U/C
**Section 3. Care and care coordination model**			
Care and care coordination model forecasts for the next 10 years	Desired	Actual	
9. The model must contemplate the creation of a hospital oncology care network and concentrate diagnostic-therapeutic activities, as well as optimising care resources	**Yes (89%)**	Yes (57%)	C/D
10. The model must promote the establishment of a nationwide population-based cancer register	**Yes (96%)**	**Yes (83%)**	C/C
11. The role of nursing in the care of LCS patients will become more important	**Yes (99%)**	**Yes (84%)**	C/C
12. The LCS patient care process will involve multidisciplinary care	**Yes (100%)**	**Yes (87%)**	U/C
13. An EPI (extended programme on immunisation) covering all levels of care will be defined	**Yes (99%)**	Yes (77%)	C/M
14. The model should consider increased collaboration between medical oncology and primary care	**Yes (100%)**	**Yes (84%)**	U/C
15. The clinical management of LCSs will have primary care as its focus	**Yes (88%)**	No (60%)	C/D
16. All LCS patients will have a case manager who will coordinate the different concurrent services and act as an accessible point of reference	**Yes (94%)**	No (69%)	C/M
17. Single points will be set up for appointment scheduling and for the collection of results in order to improve the interactive nature of care procedures	**Yes (95%)**	No (61%)	C/D
**Section 4. Clinical and care services**			
Forecasts for the next 10 years with regard to the development of clinical care protocols and cancer treatment decisions			
18. Protocols must be stratified according to LCS patient needs (chronicity, comorbidity, fragility, etc.) and risks (survival, progression-free, quality of life, etc.)	**Yes (96%)**	**Yes (91%)**	C/C
19. The protocols must consider early detection and the addressing of common problems in LCS patients (asthenia, pain, depression, etc.)	**Yes (99%)**	**Yes (95%)**	C/C
20. The protocols must emphasise the early detection of relapses and second tumours	**Yes (99%)**	**Yes (87%)**	C/C
21. The protocols will include criteria for detecting the adverse effects of treatments, their possible sequelae and iatrogenesis in general	**Yes (100%)**	**Yes (86%)**	U/C
22. It is essential that the communication process between the patient, the acute centre, primary care and social and health centres will improve	**Yes (100%)**	**Yes (83%)**	U/C
23. Therapeutic decision making must consider the specific characteristics of each patient, the risk of toxicity associated with each patient's systemic conditions at all times and the patient's own expectations	**Yes (100%)**	**Yes (95%)**	U/C
24. Therapeutic decision-making support algorithms will be required based on the LCS patient's clinical presentation	**Yes (96%)**	**Yes (94%)**	C/C
25. National *benchmarking* for decision making will be improved	**Yes (97%)**	No (59%)	C/D
Oncology service portfolio and core common portfolio forecasts for the next 10 years			
26. The clinical needs of LCSs will require redesigning the service portfolio for oncology services	**Yes (94%)**	**Yes (86%)**	C/C
27. Care circuits in A&E will have to be set up to make it possible to consider referring LCS patients to the oncology service based on the reason for consultation	**Yes (92%)**	Yes (56%)	C/
28. Including social workers in oncology services is key to providing the LCS patient with appropriate care	**Yes (86%)**	No (56%)	C/D
29. Including palliative care specialists in oncology services is key to providing LCS patients with appropriate care	**Yes (91%)**	**Yes (84%)**	C/C
30. Psycho-oncology will play a decisive role in the perception of quality of life by LCS patients and their relatives and/or the context in which they live	**Yes (94%)**	**Yes (80%)**	C/C
31. Cancer care will be increased for LCS patients	**Yes (93%)**	**Yes (84%)**	C/C
32. Comprehensive Geriatric Assessment will be a useful tool in the care of elderly LCS patients	**Yes (96%)**	**Yes (90%)**	C/C
33. The development of care pathways between oncology and home care professionals will be a solution for alleviating congestion in hospitals and for improving cancer education for LCS patients and the context in which they live	**Yes (87%)**	No (72%)	C/M
34. The home pharmaceutical service will be made more general, with the dispensing of drugs to the LCS patient's home (*home delivery*)	**Yes (81%)**	No (70%)	C/M
**Section 5. Funding model**			
Forecasts for the next 10 years with regard to the availability of resources and the funding of LCS care	Desired	Actual	
35. Resource limitation will complicate the selection of therapeutic alternatives for certain types of LCS patients	**No (82%)**	**Yes (81%)**	C/C
36. Cost–benefit analysis will be used for therapeutic decision making	**Yes (91%)**	**Yes (80%)**	C/C
37. Acute beds will need to be converted at medium/long-stay hospitals for optimising LCS patient care resource management	**Yes (89%)**	No (51%)	C/
38. Rural home care management will need to be optimised to enable efficient home hospitalisation	**Yes (98%)**	Yes (62%)	C/D
39. For certain types of LCS patients, to define and enhance the areas of palliative care that could be extended to primary care and that in some cases is currently provided in hospital care	**Yes (96%)**	**Yes (90%)**	C/C
40. Given the epidemiological scenario and the envisaged increase in the prevalence of LCSs, new funding variables that respond to new care scenarios will be considered	**Yes (89%)**	Yes (64%)	C/D
41. Clinical and care outcomes will influence funding for oncology services	**Yes (91%)**	Yes (65%)	C/D
42. The care centre funding model will be changed to a care programme funding model	**Yes (85%)**	**No (84%)**	C/C
43. There will be a tendency to centralise the purchase of drugs and other health technologies in order to reduce their cost	**Yes (90%)**	**Yes (93%)**	C/C
Forecasts for the next 10 years with regard to the funding of drugs and other health technologies			
44. Efficacy and safety will be decisive in determining the basis for funding new drugs and other health technologies in some LCS patient categories	**Yes (97%)**	**Yes (80%)**	C/C
45. The cost-effectiveness criterion will be used to determine the funding of new drugs and other health technologies in some LCS patient categories	**Yes (91%)**	**Yes (94%)**	C/C
46. The cost of treatments will be determined in accordance with their value in terms of health outcomes in some LCS patient categories	**Yes (89%)**	**Yes (83%)**	C/C
47. Investment/disinvestment criteria for drug and other healthcare technology funding will be applied in some LCS patient categories	**Yes (86%)**	**Yes (81%)**	C/C
**Section 6. Assessment measures**			
Forecasts for the next 10 years with regard to care process assessment measures	Desired	Actual	
48. LCS-specific efficacy and safety indicators will be used	**Yes (97%)**	**Yes (86%)**	C/C
49. Indicators will be defined to evaluate the entire LCS care process (primary care, hospital care, home care) in an integrated way	**Yes (98%)**	Yes (62%)	C/D
50. Sufficient means will be made available for carrying out an evaluation of the indicators	**Yes (97%)**	**No (81%)**	C/C
51. The health results obtained by each hospital in relation to LCS patients will be published regularly	**Yes (98%)**	**No (80%)**	C/C
52. Evaluation results will be used in care decision making	**Yes (99%)**	No (57%)	C/D
**Section 7. Training of health professionals**			
Health professional training forecasts for the next 10 Years	It is important		
53. Training all healthcare professionals involved in the care process with regard to the needs of LCS patients throughout the process	**97%**		C
54. Joint training of oncologists, nurses, hospital pharmacists and primary care specialists	**95%**		C
55. Specific training in LCS patient safety taking into account the comorbidities associated with these patients	**97%**		C
56. Shared decision-making training (for the entire care process) for all healthcare professionals	**94%**		C
**Section 8. LCS Patient involvement**			
Forecasts for the next 10 years with regard to the role of the LCS patient in the care process	Desired	Actual	
57. The normalisation of the LCS patient as a chronic patient will be decisive in optimising their quality of life	**Yes (96%)**	**Yes (81%)**	C/C
58. Awareness-raising campaigns will be carried out to destigmatise the LCS patient, especially for certain types of cancer	**Yes (92%)**	**Yes (84%)**	C/C
59. The involvement of patients (and their carers) in the design and development of clinical care processes and protocols will be key for ensuring their suitability	**Yes (96%)**	Yes (73%)	C/M
60. The patient will be involved in shared decision making at different points in the care process	**Yes (98%)**	**Yes (88%)**	C/C
61. Patient associations will be encouraged to contribute to empowering the LCS patient	**Yes (92%)**	**Yes (90%)**	C/C
62. LCS patients and their close family will be trained with a view to promoting healthy lifestyles	**Yes (100%)**	**Yes (95%)**	U/C
63. LCS patients will be trained for involvement in the management of their disease	**Yes (98%)**	**Yes (92%)**	C/C
**Section 9. ICT functions**			
ICT forecasts for the next 10 years	Desired	Actual	
64. Telemedicine (two-way e-health platforms) will be widely used to facilitate communication and the transmission of information during all healthcare processes between different professionals and between professionals and LCS patients	**Yes (95%)**	**Yes (94%)**	C/C
65. An integrated computerised medical record will be available for medical professionals at all levels of care provided to LCS patients, including an alert system to avoid duplication	**Yes (99%)**	**Yes (92%)**	C/C
66. Computer systems will be available for integrating clinical databases and for the creation of shared databases between various hospitals (Big Data) in order to generate evidence through real practice (*Real-World Evidence*)	**Yes (99%)**	**Yes (85%)**	C/C
67. ICTs will be available to monitor compliance with the LCS patient's prescribed treatment	**Yes (98%)**	**Yes (87%)**	C/C
**Section 10. Clinical and translational research**			
Research forecasts for the next 10 Years	Desired	Actual	
68. To have up-to-date information on ongoing clinical trials and patient selection criteria	**Yes (91%)**	Yes (70%)	C/M
69. To conduct more research on the impact patient involvement has on the care process	**Yes (89%)**	No (52%)	C/D
70. To conduct cost-effectiveness studies on new treatments	**Yes (90%)**	**Yes (89%)**	C/C
71. To conduct LCS patient quality of life studies	**Yes (95%)**	**Yes (84%)**	C/C
72. To conduct LCS patient treatment compliance studies	**Yes (93%)**	Yes (78%)	C/M
73. To conduct studies on the extent to which the recommendations in the Clinical Practice Guidelines are implemented by heath care professionals	**Yes (87%)**	Yes (52%)	C/D
74. To conduct studies on the side effects of drugs in actual clinical practice	**Yes (97%)**	**Yes (81%)**	C/C
75. To conduct research on mechanisms that allow the personalisation of treatments	**Yes (96%)**	**Yes (86%)**	C/C
**Section 11. Access to new therapies**			
Forecasts for the next 10 years with regard to access to new therapies	Desired	Actual	
76. Access to molecular diagnosis will be rolled out for all LCS patients	**Yes (95%)**	No (62%)	C/D
77. The use of the same diagnostic and technological resources will be guaranteed regardless of the LCS patient's point of access to the healthcare system	**Yes (100%)**	No (76%)	U/M
78. Access to genetic advice for informed decision making will be provided for all Spanish National Health Service users	**Yes (97%)**	No (53%)	C/D
79. The system will establish technical criteria for prioritising access to genetic counselling	**Yes (95%)**	**Yes (85%)**	C/C
80. Shared risk strategies between management and industry will address the policy of greater access to therapeutic innovation and the drive for continuous research into therapeutic innovation	**Yes (94%)**	No (56%)	C/D
**Section 12. Barriers to the transformation process**			
Barriers to be overcome during the transformation process	Difficult to overcome		
81. Healthcare professionals' resistance to change	**79%**		C
82. Decentralisation of the Spanish National Health Service	**86%**		C
83. The tendency to work individually in each specialty and/or at each level of care	**87%**		C
84. Lack of support from hospital and primary care management	**83%**		C
85. Lack of political and institutional support	**90%**		C
86. Lack of financial resources	**94%**		C
87. Lack of studies and functional population records (*Real-World Data, Real-World Evidence*) with specific LCS protocols	**89%**		C
88. Lack of social awareness of the existence of LCS patients	67%		M
89. Stigmatisation of cancer for certain types of cancer	62%		D
90. Lack of tools to assess the actual needs of the LCS patient for the purpose of defining the patient-based care model	**86%**		C

Values in bold print indicate consensus. U: unanimity; C: consensus; M: majority; D: difference of opinion

(1) Unanimity: 100% expert panel agreement; (2) Consensus: 80–99% agreement; (3) Majority: 66–79% agreement; (4) Difference of opinion: agreement < 66%

Items were responded to on an either/or basis (yes/no) or using the Likert scale (5 and 4 levels of agreement in the first and second Delphi rounds, respectively)

### Oncological epidemiology

The majority of experts (98%) were of the view that the population's access to health services was a significant factor in the prevalence of LCS. In the opinion of more than 95%, lifestyle changes and therapeutic and technological improvements will influence the number of LCS patients over a period of 10 years. Ninety-two per cent of participants stated that comorbidities in LCS patients will impact the emergence of new subpopulations and increase the complexity of care.

### Care model

There was unanimous desire that the care model for LCS patients be multidisciplinary and that oncology planning address access to personalised therapies, that LCS patients be included in care plans and that cooperation between primary care and hospital care be improved. Ninety-eight per cent of those taking part were in favour of correcting territorial inequalities through an appropriate allocation of resources, although in the opinion of the majority (71%) this situation will not be resolved in the next 10 years.

Of all the strategies proposed for improving the coordination of different levels of care, only collaboration between medical oncology and primary care was considered feasible by the consensus (84%).

### Clinical and care services

According to the vast majority of participants, protocols need to be stratified in accordance with the needs and risks of LCS patients, and there was agreement on the need to include early detection and addressing of the most common problems experienced by LCS patients, as well as relapses or the emergence of new tumours (99% in both cases).

Regarding therapeutic decisions, the need to consider patient characteristics, expectations and the risk of toxicity was unanimously accepted. National *benchmarking* (or comparative evaluation) and supporting algorithms (96% of respondents) were also recognised as appropriate strategies, but the first was not considered feasible. Only 11% of oncologists (compared to 41% of all participants) were of the opinion that this measure will be implemented in the next 10 years.

There was agreement on the need to create pathways for the emergency referral of oncology patients to the oncology department (92%), as well as the need to incorporate social workers (86%) and palliative care specialists (91%). Consensus was reached regarding the role of psycho-oncology in the perception of improvement of the quality of life of LCS patients (94%). The experts believe that healthcare links need to be established between oncology and home care professionals (87%), and home pharmaceutical dispensing (81%) needs to become more widespread, although less than one third considered these changes feasible within 10 years. Only 8% of the Health Policy group (compared to 28% of the total) were confident that these links would be established in the next 10 years.

Regarding home pharmaceutical dispensing, the hospital pharmacy group (44%) expressed less of a desire for the measure to be implemented (compared to 81% of the total), unanimously disagreeing on its feasibility.

### Funding and evaluation

More than 80% of respondents predicted that resource constraints will affect the selection of therapies for certain types of LCS patients. There was agreement on the importance of using cost–benefit analysis in decision-making (91%) and its feasibility in the future.

Panel members agreed that clinical care outcomes should influence the funding of cancer services (91%). Eighty-five per cent expressed the desire that a funding model be adopted based on care programmes rather than on centres, although almost the same percentage dismissed this as a possibility. In contrast, the centralised purchasing of drugs and health technologies to reduce costs was deemed necessary (90%) and feasible (93%).

All the proposed evaluation measures were considered necessary by virtually all participants. Eighty-six per cent were of the view that such indicators would be used in the future, although there was no consensus that the process would be evaluated in an integrated manner.

### Training of health professionals. ICT

All items presented regarding the training of health professionals involved in the care of LCS patients were accepted on a consensus basis. Mention should be made of joint training between oncology, nursing, pharmacy and primary care (95%); training in safety parameters and comorbidities in relation to LCS patients (97%); or shared decision-making (94%).

Ninety-five percent of the experts advocated the use of telemedicine to facilitate communication during the care process and between different professionals and patients; the need to implement electronic medical records (99%) and to integrate clinical databases and *big data* to generate evidence in real clinical practice (99%), in addition to using ICT to monitor LCS treatment adherence (98%).

### Patient involvement

According to a large majority (92–100%), LCS patients must be regarded as normal chronic patients, that they be involved in protocol design and shared decision-making, and that they be trained in healthy lifestyles and disease management. There was no consensus regarding patients' involvement in the design of processes and protocols (73%), but it was felt that the rest of the measures will be feasible within 10 years (81–95% agreement).

### Clinical and translational research

Ninety-one percent of panel members expressed a desire for up-to-date information on ongoing clinical trials, but no consensus was reached on the likelihood of this happening. Agreement was reached on the following research objectives: studies on the impact of patient involvement in the care process (89%), the cost-effectiveness of new therapies (90%), quality of life (95%), treatment adherence (93%), adoption of the recommendations contained in the clinical practice guidelines (GPC) (87%), the side effects of treatments observed in clinical practice (97%) and the mechanisms that make it possible to personalise LCS treatment (96%).

### Access to new therapies

Among the measures for access to new therapies, 95–100% of panel members expressed their desire for the following to be carried out within 10 years: access to molecular diagnosis, use of the same diagnostic and technological resources regardless of the LCS patient's point of access to the Spanish Health Service, access to genetic advice for making decisions using technical criteria, and the implementation of risk-sharing strategies between the public authorities and the pharmaceutical industry. Of all these measures a consensus agreement (85%) was reached only on the feasibility of defining technical criteria for prioritising access to genetic counselling.

### Barriers to the transformation process

The lack of economic resources was cited as the most difficult barrier to overcome, which 74% of participants considered insurmountable. Other barriers agreed on by more than 80% of the participants were: resistance to change on the part of health professionals, decentralisation of the Spanish Health Service, lack of coordination between specialties, or shortcomings in the support provided by hospital and primary care management, political and institutional support, functional population studies and records or tools for assessing LCS needs.

## Discussion

In this study, more than 250 oncology professionals, both in the healthcare and management fields, as well as patients, have outlined guidelines that could serve to optimise the healthcare management model in Spain so that it could be adapted to provide efficient LCS patient care. According to the experts, the portfolio of services for optimal LCS care will require reorientation and perhaps even redesign.

### Oncological epidemiology

The study identified therapeutic and technological improvements as a factor that might impact the prevalence of LCS patients in the next 10 years. The emergence of new therapies other than conventional chemotherapy is also a factor to be taken into account with regard to an increase in the prevalence of LCS patients [15], and cancer planning must address fair access to these personalised therapies.

A change in population lifestyle was another factor identified as determining the prevalence of LCS patients. However, although the benefits of this change have been described in cancer patients [16–21], the published results are limited and not entirely conclusive [1].

Most participants considered it necessary to address the correction of territorial inequalities through an appropriate allocation of resources, although doubts were expressed that this would occur in the next 10 years. Patients were particularly pessimistic on this point. Population access to health services is likely to affect the prevalence of LCS patients over the next few years, so territorial inequalities in care for LCS patients must be identified and service planning must be backed up to avoid duplication and inequalities [14]. Access to health information by disadvantaged people is an area for improvement [22–24].

In this context, one of the main challenges arising from this study is to manage the care priorities of LCS patients in accordance with their oncological and clinical presentation and with their associated comorbidities. Four out of ten cancer patients have at least one other chronic disease and 15% have at least two [25]. The existence of comorbidities substantially affects treatment decisions and the results obtained in the treatment of cancer [26], so patients will have to be stratified according to their comorbidities so that they can be treated individually.

### Care model

The need for collaboration between medical oncology and primary care was unanimously agreed, with a broad consensus that this collaboration will be strengthened in the next 10 years. Primary care must become the cornerstone for the clinical management of LCS patients, although at the same time it is questioned whether this will be a reality in the next decade due partly to the current fragmentation of the health system.

Other factors mentioned by experts that are key in addressing the relationship between primary care and hospital care need to be highlighted: the reorientation and training of professionals before they assume new roles; identification and differentiation of the characteristics of those LCS patients who require primary care and those requiring hospital care; resolving the primary care overload and the resistance of oncologists to losing LCS patients in follow-up.

There are different LCS patient monitoring models. Exclusive oncology care may have drawbacks, such as patients rejecting follow-up at the same facility where they were treated in their acute phase, or relying excessively on those facilities to address any health problems. In addition, the handling of non-cancer problems in oncology departments is often limited [27]. In community-based models, however, cancer care recommendations from primary care departments may result in less follow-up, and primary care professionals need to be specifically trained in cancer survivor care [27]. Nevertheless, the role of primary care physicians in such patients has been widely recognised [28–30].

LCS patient care appears to be based on a shared, multidisciplinary approach between primary care and hospital care [28–32]. This collaboration has proven to improve clinical outcomes, hence its consideration as a key factor in the cancer care model [33, 34]. Experts almost unanimously expressed the need to improve nursing care for LCS patients.

The study highlighted the need to redesign the LCS service portfolio, enhancing the role of psychologists, social workers and geriatricians. The importance of home care, both clinical and pharmaceutical, was also emphasised and although there was agreement on the need for this, the experts considered its viability over the next 10 years to be low. Particularly sceptical were the group of health policy participants (regarding clinical home care) and the hospital pharmacy (regarding drugs).

### Funding and evaluation

Regarding the current funding model, the panel members agreed on the relevance of considering new variables (such as results-driven or care programme funding), although it was not agreed that these changes would take place within 10 years.

LCS patient care involves both direct and indirect long-term costs [35]. In fact, it is estimated that about 25,000 people in Spain may find themselves in a vulnerable position due to the economic impact of the diagnosis and course of cancer [36]. The increase in the prevalence of LCS patients, resulting in an increase in the age of patients and their comorbidities [14], poses a challenge for the funding model.

On the other hand, this study has shown that participants perceived the design and use of indicators as a difficulty. This aspect is another challenge, since the multidisciplinary approach to LCS patient care will require shared indicators for evaluating and ensuring continued care, avoiding duplication of interventions and facilitating the patient's transition between different levels of care [37].

### Training of health professionals: ICT

This study has highlighted agreement on the importance of training all personnel involved in LCS care, beyond medical oncology. Shared decision-making and patient safety based on their comorbidities must be part of such training. Constant innovation in oncology requires the ongoing training of professionals, which also includes the patient [38, 39].

Experts agreed on the need and feasibility of implementing ICT in certain care processes. These can generate evidence in actual clinical practice and improve patient care [37].

### Patient empowerment

This study has demonstrated agreement on measures aimed at increasing the training of the LCS patient, as well as his or her collaboration in informed decision-making. It has been seen as important that patients and caregivers be provided with quality training in the areas of pathology, self-care and healthy lifestyle habits. However, there was no consensus regarding patient and caregiver involvement in the development of care protocols and processes. Such involvement would provide a better insight into what patients need from those processes, even though not all of them can take an active part in decision-making.

### Clinical and translational research

The possibility of further research on LCSs (long-term cancer survivors) in the future elicited a diversity of views, probably due to the low level of funding for this purpose. The experts agreed on the importance of carrying out cost-effectiveness studies on treatments, LCS quality of life and therapeutic adherence, and most agreed that these types of studies will be part of the research agenda in the coming years.

Clinical trials are a feature of the routine of most oncology departments [40]. However, most research resources are geared towards finding new molecules to slow the progression of the disease. Including real-life clinical practice studies in the decision making process can fill gaps in knowledge due to the inherent characteristics of clinical trials [41], although it poses a serious challenge for regulators and assessors, given the scant guidance on how to generate and integrate it efficiently. Research on LCSs could bring about an understanding of the mechanisms underlying the sequelae they suffer, demonstrate the effectiveness of interventions aimed at reducing these sequelae, or lead to the design of less toxic treatments; in addition to providing real-life data^42^.

### The transformation process and its barriers

The Delphi questionnaire concluded with a series of statements regarding potential barriers during the transformation process. Of the ten barriers identified, there was a difference of opinion in only one of these.

By grouping the barriers according to the classical understanding of management areas, those affecting macro-management gained a high consensus: lack of financial resources, lack of political and institutional support and decentralisation of the Spanish National Health Service. Second, barriers that can be located in the area of middle-level management were identified: lack of support from hospital management, tendency to work individually in each specialty or level of care, lack of studies, records and protocols; or lack of tools for assessing LCS needs. Finally, a barrier was identified at the micromanagement level (also a very significant one, especially when far-reaching transformation is the objective), that of resistance to change among professionals.

The limitations of this study are the result of its Delphi design. This methodology does not allow for the details of the items to be specified, although, when discussing the results, the comments that the panel members made on each of them were taken into account. In addition, given the approach taken for the study, it was decided to accept a broad definition of the term LCS, mentioned at the beginning of the questionnaire, which could include highly differentiated clinical profiles. However, in only two instances did participants include comments on this definition. The natural tendency to confuse forecasts (actual) with the desire that the forecast becomes a reality, which can vary from one expert to the next, may also be considered a possible limitation. Lung cancer specialists were over-represented in this study. A sub-analysis of these with respect to all oncologists resulted in significant differences greater than 20% in only eight questions, making bias unlikely. Finally, the potential influence of the study's sponsors was minimised by their not participating in the analysis, interpretation of the results or drafting of the final article.

The high degree of consensus reached in this study, more than 80% of the items and the high level of participation by the panel members, confer significant validity to the results obtained. This supports the results of this study as a road map for the transformation process to be carried out by the Spanish National Health Service in the area of LCS care. It has been possible to identify the obstacles that must be overcome, and above all the actors involved in the development of the model and their responsibilities.

Ultimately, the magnitude of the challenges posed in this study can be measured by the wide spectrum of barriers to be overcome at all levels in the health system, and because these affect all those involved in health care in our country. The purpose of initiatives such as this study is to contribute to the awareness of all those involved, in addition to providing the means for transforming the model in the medium term, so that it can develop efficiently towards better care for LCS patients.

## Conclusions

First, despite the Spanish National Health Service facing an increase in the incidence of LCSs, it would appear difficult to overcome territorial inequalities.

The role of primary care in the clinical management of LCS patients’ needs to be upgraded, and coordination with the oncologist and with hospital care is essential. The portfolio of services for optimal LCS care would require reorientation or even redesign. To this end, multidisciplinary care will need to be promoted, including nursing, psycho-oncology, palliative care, social work or pharmacy care, among other services.

The funding model needs to be adapted to the new epidemiological, efficacy and safety scenario in order to determine the funding conditions for new drugs and technologies. There is still a high level of uncertainty about how to handle this aspect, given the limitations in terms of care resources. Specific efficacy/effectiveness and safety indicators need to be used in therapeutic decision-making affecting LCSs, which must be integrated from a multidisciplinary perspective.

The new care models highlight the need for specific ongoing training for professionals involved in the care of LCSs, especially in primary care. Research in the area of LCSs is crucial, in terms of the cost-effectiveness of interventions, treatment safety, quality of life or treatment adherence; but the lack of resources will pose a barrier.

The process of adapting the care model to the increase in the incidence of LCSs presents some barriers, such as the lack of financial resources, institutional support or population studies and records.
